# Emergency Pancreatoduodenectomy with Preservation of Gastroduodenal Artery for Massive Gastrointestinal Bleeding due to Duodenal Metastasis by Clear Cell Renal Cell Carcinoma in a Patient with Celiac Artery Stenosis

**DOI:** 10.1155/2014/218953

**Published:** 2014-08-11

**Authors:** Kyriakos Neofytou, Alexandros Giakoustidis, Martin Gore, Satvinder Mudan

**Affiliations:** ^1^Upper GI/HPB Unit, Department of Academic Surgery, Royal Marsden Hospital, Fulham Road, London SW3 6JJ, UK; ^2^The London Clinic, 20 Devonshire Place, London W1G 6BW, UK; ^3^Royal Marsden Hospital, Fulham Road, London SW3 6JJ, UK

## Abstract

Duodenal metastasis from renal cell carcinoma is rare, and even rarer is a massive gastrointestinal bleeding from such tumours. Coeliac occlusive disease, although rarely symptomatic, can lead to ischaemic changes with anastomotic dehiscence and leaks when a patient undergoes pancreatoduodenectomy. A 41-year-old man with known metastasis to the adrenal glands and the second part of the duodenum close to the ampulla of Vater from clear cell renal cell carcinoma was admitted to our department due to massive gastrointestinal bleeding from the duodenal metastasis. Endoscopic control of the bleed was not possible, while the bleeding vessel embolization was able to control the haemorrhage only temporarily. An angiography during the embolization demonstrated the presence of stenosis of the coeliac artery and also hypertrophic inferior pancreaticoduodenal arteries supplying the proper hepatic artery via the gastroduodenal artery (GDA). The patient underwent emergency pancreatoduodenectomy with preservation of the gastroduodenal artery. The patient had an uneventful recovery and did not experience further bleeding. Also the blood flow to the liver was compromised as shown by the normal liver function tests (LFTs) postoperatively. To the best of our knowledge, this is the first report of a preservation of the GDA during an emergency pancreatoduodenectomy.

## 1. Introduction

Clear cell renal cell carcinoma (ccRCC) represents 3% of human malignancies [1]. 25–50% of patients experience synchronous or metachronous metastases primarily in the lungs, bone, liver, adrenal glands, and brain. However among the rare sites of metastasis of this neoplasm (urinary bladder, epididymis, iris, thyroid, breast, pancreas, spleen, gallbladder, and ampulla) is the duodenum [2, 3]. The metastases to the duodenum can lead, although rarely, to massive upper gastrointestinal bleeding. Such cases have been described in the literature and were treated with embolization [4], local resection of the tumour with wedge resection of duodenum [5], and pancreatoduodenectomy [6]. The progress that is made in the last years in surgical techniques, as well as in the perioperative intensive care treatment, has led to a significant decrease in morbidity and most of all mortality of patients that undergo pancreatoduodenectomy. Within this context, patients have undergone emergency pancreatoduodenectomy in cases of trauma, while there is little data on its use for nontrauma patients.

Coeliac artery stenosis, although not a rare condition as described in 4–20% in the general population [7], does not often cause symptoms. This is due to the rich collateral network between superior mesenteric artery (SMA) and celiac artery. This network is mostly comprised of the pancreaticoduodenal arcades formed by the superior and inferior pancreaticoduodenal arteries as well as the dorsal pancreatic artery from the splenic artery. A significant problem, however, arises in these patients if they undergo pancreatoduodenectomy, as part of the procedure includes division of the GDA resulting in the interruption of the main route of collateral circulation between SMA and coeliac artery pancreaticoduodenal arcades. This can lead to serious postoperative complications (failure of ischemic anastomoses) due to the reduction or even interruption of the blood flow in supramesocolic viscera [8]. The above situation is not so rare, as coeliac artery stenosis is observed in 2%–7.6% of patients who undergo pancreatoduodenectomy [9, 10].

The pylorus preserving pancreaticoduodenectomy (PPPD) with preservation of GDA was first described in 1996 by Nagai et al. [11]. The main objective of this operation, when first described, was to maintain duodenal and pyloric perfusion, thereby reducing the likelihood of rupture of duodenojejunostomy. This technique lapsed as the accumulated experience of the application of PPPD showed that duodenojejunostomy is safe despite the division of the GDA.

Here, we present a rare case of a patient with massive upper gastrointestinal bleeding due to duodenal metastasis of ccRCC in whom a celiac artery stenosis coexisted. This patient underwent emergency PPPD with preservation of the GDA. To the best of our knowledge this is the first report of such a case especially as an emergency operation.

## 2. Case Report

A 41-year-old man with known metastasis to the adrenal glands and the second part of the duodenum close to the ampulla of Vater from clear cell renal cell carcinoma (ccRCC) was admitted to our department due to massive gastrointestinal bleeding.

The patient had undergone left nephrectomy one year ago for ccRCC (Fuhrman Grade 4, with vascular invasion). Six months after the nephrectomy, the patient showed progression of disease, with adrenal metastasis bilaterally and to the midthoracic lymph nodes. The patient underwent 4 cycles of pazopanib and a computed tomography (CT) scan showed good response. Unfortunately a CT after six cycles of pazopanib showed progression of disease with an increase of the size of the known metastasis and also a duodenal metastasis confirmed with biopsy.

**Figure 1:**
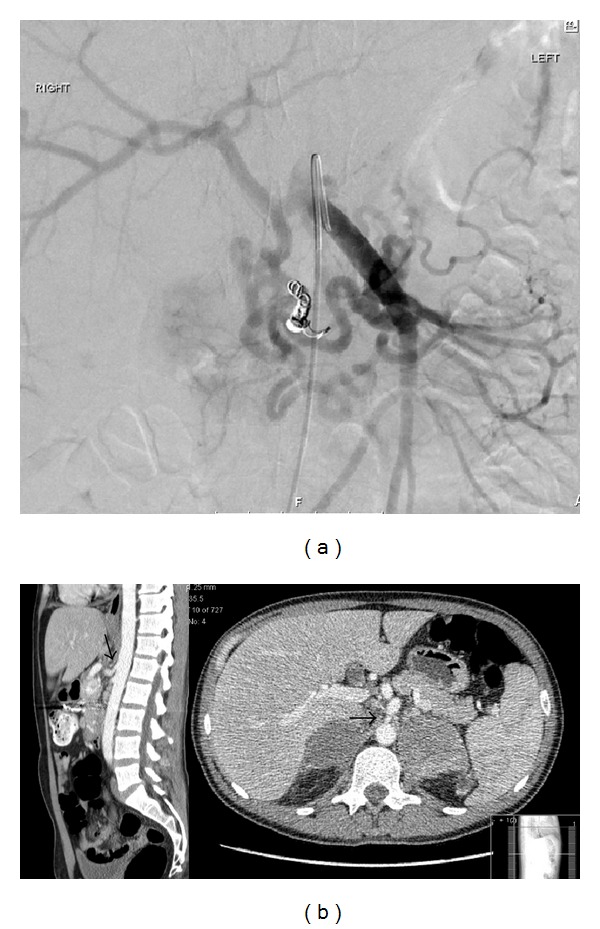
(a) Selective catheterization of SMA, showing hypertrophic inferior pancreatoduodenal arteries supplying through the GDA the proper hepatic artery and right and left hepatic arteries. Coils in posterior inferior pancreaticoduodenal artery. (b) CT scan (arterial phase). Stenosis at the origin of the celiac artery with poststenotic dilatation (arrows). Cross-sectional images illustrated the sizeable bilateral adrenal metastases.

Following emergency admission, endoscopy revealed the site of haemorrhage to be the known metastasis at the second part of the duodenum near the ampulla of Vater. Endoscopic haemostasis was not possible and the patient underwent angiography with a plan for embolization. The angiography revealed the presence of stenosis of the coeliac artery and also hypertrophic inferior pancreaticoduodenal arteries supplying through the GDA the proper hepatic artery (Figure 1(a)). The embolism of the bleeding vessels (superior and inferior posterior pancreatoduodenal arteries) controlled only temporarily the haemorrhage with the patient deteriorating 24 hours later.

In this interval the patient had a CT of chest/abdomen/pelvis for staging of the disease, which confirmed the findings of the angiography in regard to the stenosis of the celiac artery and a dilatation distal to the stenosis (Figure 1(b)). The CT also showed extended progression of the disease with an increase of size of the adrenal metastasis (the right one from 3.2 cm to 5.2 cm and the left one from 7.1 cm to 7.9 cm), an increase of size of the known duodenal metastasis (from 2.5 cm to 4.5 cm), and also five new liver metastases with the bigger one in segment VI measuring 12 mm, while there was progression of disease in the thorax with an increase of the midthoracic lymph nodes and development of lung metastasis with extensive pleural effusions.

The deterioration of the haemorrhage led to a haemodynamically unstable patient who was taken to theatre. The extent of the metastatic disease necessitated a palliative surgical intervention in order to control the bleeding and stabilize the patient, with a plan for second line chemotherapy or participation in a clinical trial.

The fact that the bleeding duodenal tumour was very close to the ampulla of Vater, reconfirmed with intraoperative endoscopy prior to laparotomy, made a PPPD the only choice.

**Figure 2:**
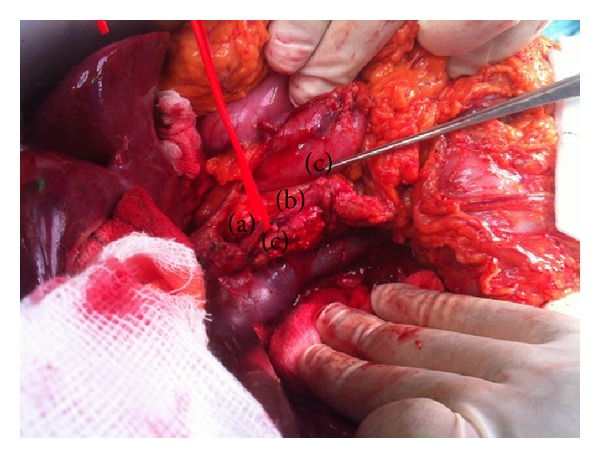
Pancreatoduodenectomy with preservation of GDA. (a) GDA, (b) anterior pancreaticoduodenal arcade, and (c) stumps of superior posterior and inferior posterior pancreaticoduodenal arteries.

During laparotomy, the presence of sizeable adrenal metastasis bilaterally was confirmed, as well as the metastasis in the duodenum, in the para-aortic lymph nodes, while the intraoperative ultrasound demonstrated five metastatic sites in the liver. Following mobilization of the duodenum and the formation of a tunnel between the pancreas and the SMV/portal vein, the GDA was dissected. The occlusion of the GDA led to the vanishing of pulse in the common, right, and left hepatic artery, confirming the findings of the angiography with the arterial flow to the liver based on a collateral network between the SMA and the hepatic artery via the GDA. This led to the decision for a PPPD with preservation of the GDA. The posterior superior pancreatoduodenal artery, a branch of the gastroduodenal artery, and the posterior inferior pancreatoduodenal artery, a branch of the SMA, as shown on the angiography that they gave blood supply to the duodenal metastasis, were dissected and ligated with established presence of pulse in the hepatic artery following this. On the contrary the branch of the gastroduodenal artery and the arterial network between superior and inferior anterior pancreatoduodenal artery were preserved (Figure 2). The PPPD was completed without further modifications and with three anastomoses in the same loop of jejunum, an end-to-side pancreaticojejunal (duct to mucosa), end-to-side hepaticojejunal in a retrocolic fashion, and end-to-side duodenal-jejunal in antecolic fashion. The reconfirmation of presence of pulse in the hepatic artery after the end of the operation confirmed the blood flow from the SMA to the hepatic artery via the GDA.

The patient was transferred to the intensive care unit. The patient's recovery was complicated by a surgical wound infection requiring the use of a vaccum system which led to longer in-hospital stay. There was no bleeding following the surgical intervention and also the LFTs during the in-hospital stay were normal showing an adequate blood flow to the liver.

The histopathology test of the specimen confirmed the duodenal tumour to be a metastasis from the known ccRCC (immunohistochemistry showed that the tumour cells strongly express PAX8, RCCAg, AMACR, and CD10 with more focal expression of E-cadherin, C2M5.2 vimentin, and EMA). There was no tumour expression of CD117 or CK7. This immunoprofile which was consistent with a metastatic clear cell renal cell carcinoma was similar to that seen in the original kidney tumour. Twenty-four out of twenty-eight resected lymph nodes were infiltrated with metastasis. The tumour was completely excised (R0).

The patient was discharged twenty-six days after the operation; however, further systematic therapy was not possible as the patient died two months later due to a generalized extensive disease to the lungs, the brain, the liver, and the bones.

## 3. Discussion

The haemorrhage from a metastatic tumour to the duodenum from ccRCC, although infrequent, is well reported in the literature [5, 6]. Such emergency cases have been described as case reports and their management varies from embolization of the bleeding vessels to a surgical intervention [4–6].

In general the great vascularity of these tumours makes the endoscopic haemostatic control problematic while at the same time it decreases the effectiveness of the intravascular attempts. The above characteristics sometimes lead to an emergency operation for these patients with a main target of controlling the bleeding. Our patient was not an exception to these with the endoscopic and intravascular haemostasis not offering a permanent result of control of the bleeding.

The urgency of the situation, as the patient had a further haemorrhage along with the fact that the initial intravascular attempt for haemostasis offered a temporary effect, led us to the decision that a repeat attempt for celiac artery dilatation which could potentially offer a better access to the bleeding vessel would only result in loss of valuable time.

While the target of such surgical interventions is saving the life of a haemodynamically unstable patient, the oncological surgical approach of the metastatic disease is not a priority. This fact adds to an approach of a more conservative operation and also to a local resection of the bleeding tumour with partial duodenal resection. Such an approach has a significantly smaller morbidity and mortality when compared to pancreatoduodenectomy in such haemodynamically unstable patients. Although such an operation would be ideal for our patient, the tumour's close proximity to the ampulla of Vater and the tumour's size (4.5 cm) made this approach almost impossible. On the other hand the extended metastatic disease in conjunction with haemodynamical instability made the pancreatoduodenectomy of very high risk with the expected benefit regarding long term survival being relatively small given the progression of disease while on chemotherapy. However the fact that the patient was young along with his determination led to the decision of a pancreatoduodenectomy with the target of achieving haemostasis and stabilization of the patient so that he could have further course of chemotherapy.

The only alternative to PPPD was the surgical ligation of the vessels supplying the tumour (the posterior pancreatoduodenal artery and the posterior inferior pancreatoduodenal artery). Indeed these vessels were dissected and ligated prior to the pancreas being transacted. The decision to remove the tumour by a PPPD was based on the following. (1) Although the tumour did not cause clinical symptoms of obstruction (obstruction of the duodenum or obstructive jaundice), both the size and position of the tumour, as it was very close to the ampulla of Vater, indicated that the occurrence of such symptoms could take place sooner than later. Therefore if the tumour was not resected, a gastric and biliary bypass would take place. (2) The surgical ligation of the vessels supplying the tumour could potentially result in postoperative necrosis and perforation of the tumour and such an unfavourable postsurgical complication would require a second urgent surgical intervention in a fragile patient. The above data combined with our sufficient experience in performing PPPD with low mortality rates led to the decision to proceed with PPPD.

A stenosis of the coeliac artery, as mentioned above, is not a particularly infrequent condition [7]. The categorizations for this condition are (1) intrinsic stenosis (concentric type) due to atherosclerotic disease which is the most common cause, being the cause for the stenosis in 87% of cases in western countries, (2) extrinsic stenosis (eccentric type) due to compression by the medial arcuate ligament or periarterial ganglionic tissue, and (3) other rare causes including neoplastic disease, acute or chronic dissection, external compression by an inflamed pancreas, or vascular injury [7]. Although it rarely leads to symptomatology, the management of symptomatic patients includes (1) endovascular treatment with balloon dilatation with or without stenting [12, 13], (2) arterial bypass grafting or arterial reimplantation [14, 15], and (3) medial arcuate ligament and periarterial tissue transection in cases of extrinsic pressure [16].

The possible hazard in these patients when they undergo pancreatoduodenectomy because of the resulting inhibition of collateral blood flow to organs affected by ligation of the GDA has highlighted the importance of a routine preoperative angiography in all patients undergoing pancreatoduodenectomy [17], so that the patients with stenosis of the coeliac artery can be detected and this problem can be managed prior to or during the surgery. Such an approach however was not accepted because a simple manoeuvre of stopping the blood flow of the GDA intraoperatively before the ligation and the simultaneous feel of the hepatic artery can indicate whether an arterial flow to the liver and also to the other organs connected to the branches of the coeliac artery depends on a blood flow from the SMA to the GDA [8].

In the case of our patient, the preoperative angiography done to achieve haemostasis via embolization revealed the presence of stenosis of the coeliac artery and the blood flow from the SMA to the hepatic artery via the GDA and the collaterals between the superior and inferior pancreatoduodenal arteries. This was confirmed intraoperatively as occlusion of the GDA led to lack of pulsation of the hepatic artery. The fact that it was an emergency case made an endovascular attempt for coeliac artery dilatation risky as there would be a delay of the surgery. On the other hand the serious condition of the patient made it necessary for a short surgical intervention from a time point of view and excluded the possibility of reimplantation of the branches of the coeliac artery. With all this in mind, a PPPD with preservation of the GDA and the posterior pancreatoduodenal arterial network was carried out. This led to achieving haemostasis without a great increase of the operation's duration which was three hours and also led to the preservation of the collateral network between SMA and coeliac artery resulting in a smaller risk of anastomotic leak.

## 4. Conclusion

Although a massive bleeding caused by duodenal metastasis is an infrequent condition, it should be included in the differential diagnosis when a patient presents with such symptoms and has a history of cell renal cell carcinoma. Usually prior to surgery, there has been an attempt for intravascular embolization which had failed or managed to control the haemorrhage only temporarily. This would also reveal a possible feature of stenosis of the coeliac artery, allowing the surgeon to be aware about this condition before surgery. If the coeliac artery stenosis has not been treated during the angiography successfully, as this approach may be the most appropriate for these patients, the PPPD with preservation of the GDA, as presented in this case report, would be more appropriate for the management of such conditions.
